# Protein-Based Salivary Profiles as Novel Biomarkers for Oral Diseases

**DOI:** 10.1155/2018/6141845

**Published:** 2018-11-07

**Authors:** Alejandro I. Lorenzo-Pouso, Mario Pérez-Sayáns, Susana B. Bravo, Pía López-Jornet, María García-Vence, Manuela Alonso-Sampedro, Javier Carballo, Abel García-García

**Affiliations:** ^1^Oral Medicine, Oral Surgery and Implantology Unit, Faculty of Medicine and Dentistry, Universidade de Santiago de Compostela, Health Research Institute of Santiago de Compostela (IDIS), Santiago de Compostela, A Coruña, Spain; ^2^Proteomic Unit, Health Research Institute of Santiago de Compostela (IDIS), Santiago de Compostela, A Coruña, Spain; ^3^Department of Oral Medicine, Faculty of Medicine, Regional Campus of International Excellence “Campus Mare Nostrum”, University of Murcia, Espinardo, Murcia, Spain; ^4^Department of Internal Medicine and Clinical Epidemiology, Santiago de Compostela University Hospital Complex (CHUS), Health Research Institute of Santiago de Compostela (IDIS), Santiago de Compostela, Galicia, Spain; ^5^Department of Food Technology, Faculty of Sciences, University of Vigo-Ourense Campus, Ourense, Spain

## Abstract

The Global Burden of Oral Diseases affects 3.5 billion people worldwide, representing the number of people affected by the burden of untreated dental caries, severe periodontal disease, and edentulism. Thus, much more efforts in terms of diagnostics and treatments must be provided in the fight of these outcomes. In this sense, recently, the study of saliva as biological matrix has been identified as a new landmark initiative in the search of novel and useful biomarkers to prevent and diagnose these conditions. Specifically, saliva is a rich reservoir of different proteins and peptides and accessible due to recent advances in molecular biology and specially in targeted and unbiased proteomics technologies. Nonetheless, emerging barriers are an obstacle to the study of the salivary proteome in an effective way. This review aims at giving an overall perspective of salivary biomarkers identified in several oral diseases by means of molecular biology approaches.

## 1. Introduction

Saliva is a complex biological matrix generated by the salivary glands. Each salivary gland emits considerably different secretions with a highly variable composition depending on sympathetic and parasympathetic stimulation, circadian rhythm, eating habits, health-illness spectrum, drug intake, and other conditions [1]. The basic secretory units of salivary glands are clusters of cells called acini. The main three pairs of salivary glands in humans (parotid, submaxillary, and sublingual) together with the minor salivary glands generate 0.75–1.5 liters of this exocrine secretion per day. This physiological secretion remains high during the day, reducing significantly during the night [2].

Besides water, saliva contains a large number of electrolytes (i.e., Ca^2+^, Cl^−^, H_2_PO_4_^−^, HCO_3_^−^, I^−^, K^+^, Mg^2+^, Na^+^, and SCN^−^), proteins (i.e., mucins, enzymes, and immunoglobulins), lipids, and other molecules [3]. Saliva plays a pivotal role in the early stages of digestion, allowing a correct physiological homeostasis in human through nutrition [4]. Salivary antioxidant capacity is mainly related to some enzymes (i.e., salivary peroxidase, superoxide dismutase, catalase, glutathione peroxidase, and myeloperoxidase), uric acid, and, to a less extent, ascorbic acid and albumin [5]. In this sense, saliva is the first line of defence against oxidative stress (OE), reactive oxygen species (ROS), and free radicals [6]. Imbalance between the systemic manifestation of ROS has been implicated in the pathogenesis of over 100 pathological conditions and also in the prevailing free-radical theory of aging [7].

Recently, the term liquid biopsy (LP) was coined in analytic chemistry as the sampling and analysis of nonsolid biological tissues, primarily blood and also saliva and other biofluids. LP methodologies allow the biomonitoring of several biomarkers such as proteins, nucleic acids, circulating tumor cells, or disease drivers related to infections which proved usefulness in the diagnosis, prognosis, and staging of a large number of pathologies [8]. In principle, when saliva is compared with other biofluids (e.g., blood serum, amniotic fluid, cerebrospinal fluid, and bronchoalveolar lavage fluid), this matrix seems attractive over the others due to its noninvasive nature, its lower economic cost, and its greater clinical safety. Although certain pathologies and adverse drug reactions may limit the bioavailability of this fluid [9], saliva remains as a window of opportunity for modern medicine [10]. In this sense, this matrix has been used by medicine for the biomonitoring of physiological functions for more than a century. A good example would be salivary cortisol determinations which have been widely used in medicine and behavioural research in the last 150 years for their easy conservation and handling. Therefore, salivary cortisol is stable at room temperature for 1–2 days and at 4°C for one week [11].

Currently, several sensitive analytical techniques allow the detection and quantification of a large number of biomarkers in saliva such as mass spectrometry (MS), reverse transcription-polymerase chain reaction (RT-PCR), microarrays, nanoscale sensors, magnetic resonance spectroscopy (MRS), Western blot, immunoassay techniques, or enzymatic assays. A continuous and exponential growth in the saliva-related research lines has occurred throughout the last decades and new relevant concepts as point-of-care (POC) diagnostics have emerged [12]. In the past, cost-effectiveness analysis applied to these techniques showed them as not appropriate for clinical purposes; however, nowadays, these barriers are being effectively addressed, and this approaches are being progressively translated to clinical practice [13]. Currently, five alphabets (also known as “OMICS”) of biomarkers present in saliva are known: proteome, transcriptome, microRNA (miRNA), metabolome, and microbiome [14].

**Figure 1:**
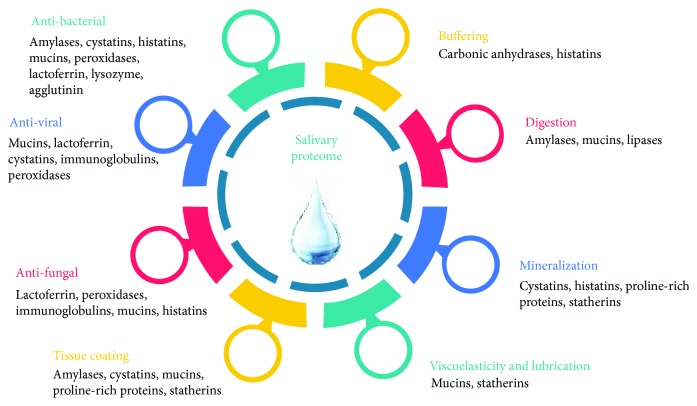
Biological function of the salivary proteome (adapted from Van Nieuw Amerongen et al. [159]).

In the field of salivanomics, the greatest advances in recent decades have focused on the analysis on nucleic acids; despite this, some interest has also been placed on protein-based techniques. Human saliva is a rich reservoir of proteins and peptides; in fact, it gathers more than 3652 proteins and 12,562 peptides and shares almost 51% of the proteins and 79% of the peptides contained in the plasma [15, 16] (Figure 1). Recent advances in proteomics techniques have brought the discovery of a large number of biomarkers and therapeutic targets in a large number of oral diseases and systemic pathologies with repercussions in the oral cavity [17]. A new landmark in salivanomics has been the discovery of the presence of exosomes and its outstanding stability in saliva. Exosomes are extracellular vesicles involved in intercellular traffic [18]. These vesicles comprise genetic material (i.e., miRNAs) and proteins. Exosomes play a pivotal role in immune system modulation, inflammation, and oncogenesis [19]. On the other hand, the discovery of the function of certain salivary peptides has helped in the development of new antibiotics [20].

In the present review, the most relevant scientific information published to date related to the salivary proteome within the spectrum of oral diseases is collected and critically discussed. This paper is mainly focused on proteins of human origin present in saliva and not on the oral disease driver-related proteins or the ones related to the pathogen-host-environment interplay.

### 1.1. Methods for Collecting Saliva

Protein kinetics and its concentrations in saliva are influenced by several factors. In this line, quantity and composition of extracted saliva are affected by the time of day, degree of hydration, body position, psychological stimuli, drug intake, health-related behaviours, systemic/oral health, and other factors [21]. In addition, deficits in sample collection, sample handling, and sample transport to the laboratory can trigger preprocessing problems. Thus, proteomic literature has extensively expressed the necessity of highly standardized protocols and tailored to fit the experimental design [22].

At this point, it is important to highlight that saliva can be collected under resting or stimulated conditions. Salivary gland stimulation can be achieved by means of different stimuli such as chewing (gums or swabs), taste stimuli (citric acid), or pharmacologic and electric stimulants [22]. Salivary flow is controlled by the autonomic nervous system. Parasympathetic stimulation produces a higher flow rate, while sympathetic stimulation produces a small flow but richer in proteins and peptides. This stimulation provides clear differences in the snapshot of the salivary proteome and also in the relative amount of specific proteins detected [23].

On the other side, saliva can be collected as whole saliva (WS) or individual gland saliva. Different approaches have been described in order to obtain single gland fluids. Regarding to parotid gland saliva, different methods can be used such as the Lashley's cup [24] or the modified Carlson-Crittenden device [25]. Submandibular and sublingual gland saliva can be collected by means of Truelove's V-shaped collector [26] or Fox's micropipette [27]. Minor gland secretions can be collected by pipettes, absorbent papers, or capillary tubes [28]. A relevant drawback in relation to the majority of these methodologies is the requirement of duct cauterization, which in practice is technically demanding and uncomfortable for patients [22].

In the case of WS, regardless of the approach used, patients should refrain from eating, drinking, and oral hygiene procedures for at least one hour before collection, and just before this process, use deionized water as a mouth rinse. Specifically, to collect unstimulated whole saliva (USWS), the patient must be kept comfortably seated avoiding orofacial movements during 5 minutes [29]. Navazesh described four approaches to collect WS: draining, spitting, suction, and the swab method. Due to the preference of collecting USWS, the gold standard method is draining [22]. Different devices have been developed in order to collect passive drool such as Salivette® (Sarstedt, Nümbrecht, Germany), Quantisal® (Immunalysis, Pomona, CA, USA), Orapette® (Trinity Biotech, Dublin, Ireland), and SCS® (Greiner-Bio-One, Kremsmünster, Austria) [30]. Several reports have shown that the protein coverage does not suffer relevant changes in relation to different collection devices. The only well-known WS drawback versus single gland saliva is that it has a higher proportion of certain nonsalivary materials such as desquamated epithelial cells, food debris, bacteria, or leukocyte in WS when compared to single gland saliva [1].

The published scientific literature on the effect of preanalytical variables on saliva profiling is scarce. Controversies are specially accentuated when the focus is put on centrifugation speed, addition of a protease inhibitor cocktail (PIC), and storage temperature range [31]. Schipper et al. demonstrated that in the case of MS-based techniques, centrifugation speed does not have an effect on the number of proteins but a small effect on the intensity of the peaks [31]. Mohamed et al. reported that centrifugation can compromise the identification and quantification of larger proteins [32]. PICs (e.g., aprotinin, leupeptin, antipain, pepstatin A, phenylmethylsulfonyl fluoride, EDTA, and thimerosal) can avoid proteolysis through the inhibition of serine-, cysteine-, aspartic-, and metallo-proteases. Nevertheless, PICs cannot fully inhibit proteolysis, and this phenomenon can occur during centrifugation especially on low-molecular-weight proteins [33]. It is worth mentioning that the addition of some reagents such as sodium azide can cause interference in immunoassays with horseradish peroxidase [33]. Despite these limitations, the majority of the described protocols use PICs to stabilize this matrix [29]. Collected samples must be collected in an ice container and proceeded in the laboratory within one hour; this methodology avoids bacterial action and minimizes posttranslational modifications (PTMs) [21]. More than 700 different species of microorganism cohabit in saliva [34]. A significant part of these microorganisms produce a variety of proteolytic and other enzymes that can trigger PTMs [29]. Moreover, temperature is known to play a pivotal role in proteostasis; for example, some proteases can function as chaperones (i.e., “helper” proteins) at low temperatures, but they act as proteases at elevated temperatures [35]. After processing, storage at −80°C have shown to provide the same spectra as fresh samples, while at −20°C temperature results can be distorted [31].

Finally, many salivary proteins of low abundance, suffer a strong interference with other more abundant proteins (i.e., lysozyme and *α*-amylase) resulting in a low ionization efficiency in MS-based analysis. There are mainly three methods for the removal of high-abundance salivary proteins: enzyme substrate absorption method used for alpha-amylase affinity removal, immunodepletion method, and the combinatorial peptide ligand library [14].

### 1.2. Analysis

Quantitative molecular biology techniques remain as the gold standard in the study of the salivary proteome [36]. These techniques are classified into absolute quantification techniques in which the exact concentration of proteins in a matrix is detected and the relative techniques in which the difference in protein concentration between samples is measured. Relative quantification techniques fit a very broad field of experimental designs; in this sense, semiquantitative ELISA, MS, and two-dimensional gel electrophoresis (2-DE) have been widely used. Nonetheless, absolute quantification approaches such as quantitative ELISA assays or multiplexed immunobead-based assay have also been used [37]. Recently, in the search for salivary biomarkers, nontargeted techniques have been successfully introduced. In this sense, the current state-of-art techniques are 2-DE techniques coupled to matrix-assisted laser desorption/ionization-time of flight mass spectrometry (MALDI-TOF MS) or liquid chromatography tandem-mass spectrometry (LC-MS/MS) [38]. Moreover, other non-gel-based approaches such as isobaric tags for relative and absolute quantitation (iTRAQ) or label-free quantification have been used for the quantitative analysis of the salivary proteome [39]. Minority, surface-enhanced laser desorption/ionization-time of flight (SELDI-TOF) MS was also used [40].

## 2. The Salivary Proteome in the Health-Illness Spectrum

### 2.1. Salivary Proteomic Profile in Health

A recent collaborative study among three reference centres in the saliva research revealed the presence of 1939 different proteins obtained from 19,474 unique peptides in whole saliva [41]. Despite this, there may be variations in this number depending on the equipment and techniques used [42]. Zhao et al. recently studied the number of matching proteins in five body fluids (i.e., plasma, urine, cerebrospinal fluid, amniotic fluid, and saliva) finding a total of 564 common proteins [43]. It has been hypothesized that the common proteins present in both plasma and saliva may be due to the intimate contact of saliva with crevicular fluid present at the periodontal pocket of sulcus level (such as albumin, transferrin, and immunoglobulins G and M) [34]. Nevertheless, several transport mechanisms capable to allow this communication have been identified such as passive diffusion, pinocytosis, and fusion pores at acinar cells [44]. Most of the salivary proteins have a low molecular weight. Specifically, 70% of the salivary proteome is made up of proline-rich proteins (PRPs) synthesized from the genome contained in chromosome 12 [45]; the rest of the proteins are synthesized from genome belonging to chromosomes 4 and 20 [46]. The salivary proteome is highly dynamic. Its proteins are affected by a large number of PTMs such glycosylation, phosphorylation, acetylation, ubiquitination, methylation, deamidation, sulfation, or proteolysis. The homeostatic mechanisms that regulate these modifications are not well known, but they constitute a particular “biological signature” not included in the genome [47]. ROS can also affect salivary proteins; in this sense, they can damage proteoglycans and can cause the oxidation of some relevant proteases. Some of these PTMs may increase the molecular weight of these proteins [48]. In addition, the salivary “interactome” of these proteins has been recently investigated. In this sense, most proteins interact with others creating protein complexes (e.g., amylase with MUC 5B, MUC 7, histatin 1, and histatin 5) [49].

Due to the limitations that the use of single OMIC technique entails, recently, they tend to be combined in order to obtain a better vision of the disease and its progression [50–53]. In this regard, current theories point to a bidirectional relationship between salivary microbiome and proteome. The salivary proteome thereby confers long-term stability to the composition and activity of the oral microbiota [50].

### 2.2. Dental and Periodontal Diseases

Table 1 summarizes the use of protein-based techniques for salivary biomarker identification in dental and periodontal diseases.

#### 2.2.1. Periodontal and Peri-Implant Diseases

The most common forms of periodontal disease are gingivitis and periodontitis. Gingivitis is defined as a plaque-induced inflammation of the marginal gingiva, whereas periodontitis (PD) implies a chronic inflammation that causes the destruction of the connective tissue of the tooth and surrounding alveolar bone [54]. PD is one of the most frequent inflammatory events in humans; in fact, one of every two Americans aged 30 or older are affected by PD (i.e., 64.7 million people) [55].

Schenck et al. demonstrated that high levels of salivary IgA were related with higher susceptibility to gingivitis when the host response to several bacteria was investigated [56]. Another nontargeted salivary proteomics research designed with Löe's concept of experimental gingivitis analysed using 2-DE found that, in patients suffering from gingivitis, there was a greater presence of serum-related proteins such as immunoglobulins and keratins in relation to the control group [57]. Nonetheless, the majority of the investigations analyse the inflammatory condition proteome in the gingival crevicular fluid and not in the saliva [39, 58]. A problem reflected in the literature regarding MS (specifically LC-ion trap MS, LC-Orbitrap MS, or LC-FTMS) is its lower sensitivity to detect certain proinflammatory and anti-inflammatory cytokines versus ELISA techniques [59]. These cytokines are very relevant in the genesis of the periodontium pathology [60, 61]. If we take a closer look at the studies that use ELISA techniques to detect different levels of proteins in saliva from patients with gingivitis compared to controls, we will find a large number of overexpressed proteins in affected subjects: TNF-*α*, IL-1, Annexin-1, HBD-1, HBD-2, HBD-3, 25-hydroxy-vitamin D3, PGE2, Cystatin C, etc. [62–66]. Due to the reversible character of this outcome, the two most used patients' subgroups in this type of research have been children and pregnant women.

Chemical studies applied in the study of PD have been constant in the medical literature for the last 70 years [67]. However, due to the lack of stable criteria and classification to diagnose this family of pathologies [68], all these investigations went through great biases until the last 30 years. PD-related salivary proteins have been classified in four subgroups [69].

The most specific salivary group biomarkers are the immunoglobulin (Ig) family proteins. Igs are glycoproteins of the *γ*-globulin type that acts at the saliva level in the identification and neutralization of bacterial agents. Immunofluorescence studies have shown that these Igs are synthesized by plasma B cells located at the level of salivary glands [12]. In this regard, countless studies have studied the differential levels of IgA, IgG, and IgM expression in control patients versus patients with different forms of PD [70, 71]. The main analytical techniques used to determine these Igs in saliva are radial immunodiffusion (RID), nephelometry, and ELISA [72]. Several studies have shown that the levels of these Igs in both chronic and aggressive periodontitis are higher than in healthy patients [69]. At the same time, it has also been shown that the level of these proteins decreases significantly with periodontal treatment. In addition, oral dysbiosis may trigger the production of specific proteases against Igs [73].

The second group comprises nonspecific markers. In this regard, there is an innumerable amount of nonspecific proteins that have been found altered in patients with periodontal disease versus healthy patients. Among them, we find, for example, albumins, amylases, mucins, lactoferrins, lysozymes, histatins, or proteins related to oxidative stress (OS). Nontargeted proteomic techniques are the most used to identify these nonspecific biomarkers [74–77]. Bostanci et al. demonstrated through label-free quantitative proteomics that patients with PD had lower levels of lactoferrin, lacritin, sCD14, Mucin 5B, and Mucin 7 vs. control [78]. This finding points to a reduction in the salivary antimicrobial and defence properties among PD-affected patients.

The third group comprises proteins related to systemic and local inflammation at the soft gingival tissues level. In this sense, C-reactive protein (CRP) and cytokines stand out. At the same time, within the group of cytokines, there are several remarkable subfamilies such as those of IL-1 (11 proteins), TNF-*α* (19 proteins), chemokines, growth factors, or bone metabolism-related cytokines (i.e., RANK/RANKL/OPG) [79].

CRP is an acute phase protein, whose levels rise in response of inflammation. This analyte can be detectable in saliva by means of ELISA [80] and integrated microfluidic platforms [81]. According to a recent systematic review, high salivary levels of CRP have been correlated with local inflammation (PD) and systemic inflammation [82]. At present, the most widely studied PD-related cytokines have been interleukin-1 beta and hepatocyte growth factor. Several case-control studies confirmed that both proteins are overexpressed in PD-affected patients vs. control [83, 84].

The RANKL/RANK/OPG pathway is responsible for controlling osteoclastogenesis [85]. Apparently, at the salivary level, high and low levels of RANKL of OPG, respectively, have being found during PD [86].

The last groups of proteins are metalloproteinases (MMPs). MMPs are a subfamily of zinc-dependent proteases responsible of extracellular matrix (ECM) remodelling. Aside from their initial role as ECM modifiers, MMPs also interact with several cell-surface molecules (i.e., chemokines, cytokines, growth factors, intercellular junction proteins, other proteases, and cell receptors). Imbalance in the ECM equilibrium has been linked to alterations at tissue remodelling, inflammatory response, cell growth, and migration [87]. Many scientific reports have given insight into MMPs and their relationship with periodontal inflammation and destruction due to the pivotal role of these proteases in collagen degradation.

The MMPs 8 and 9 are the main detectable ones in saliva. One of the actual gold standard biomarkers of PD is salivary MMP8, as several ELISA and POC platforms have ascertained [87]. Meschiari et al. demonstrated that salivary MMP9 (also known as gelatinase B) is overexpressed in PD-affected patients by zymography approaches [88].

Recent reports have used proteomic techniques in the search of salivary biomarkers in peri-implant diseases (i.e., peri-implantitis and peri-implant mucositis). These reports have shown a series of markedly overexpressed proteins in these pathological conditions, especially cytokines (i.e., IL-1b and RANK/RANKL/OPG) and MMPs (MMP8) [89]. These biomarkers are very close to those described in the PD; this finding supports the epidemiological relationship between PD and peri-implant diseases [90]. A particular proteomic signature has been also detected in the processes of root resorption induced by orthodontic movements by means of 2-DE coupled to MALDI-TOF-MS [91, 92].

#### 2.2.2. Caries

Caries is a biofilm-mediated carbohydrate-driven pathological condition. This outcome produces the mineral breakdown of the dental tissues [93]. Dental caries at permanent dentition is the most common human diseases, affecting 2.4 billion people (40% of the global population) [94]. Classically, the diagnosis of this condition has been made through conventional clinical diagnosis and radiological techniques [95]; however, recent studies at the salivary level have also served to find new useful biomarkers in the diagnosis and response to treatment of this outcome [14]. Different salivary parameters outside the proteome have been studied and correlated with the predisposition to dental caries such as dysbiosis of microbiota, evaluation of pH, buffering capacity, viscosity, and flow rate levels [96]. The biomarkers currently detected at the salivary proteome level were recently classified by Gao et al. into three subgroups: Igs, innate (nonimmune) host defence proteins and peptides, and proteins and peptides implicated on calcium phosphate chemistry.

In relation to Igs, the evidence is limited in relation to IgA and salivary IgG [97]. Nonetheless, Fidalgo et al. recently developed a meta-analysis of case-control studies to explore salivary IgA levels in dental caries concluding that high levels of IgA were higher in patients with caries (0.27 OR [0.17–0.38]) [98].

Regarding nonspecific proteins, different case-control studies with nontargeted proteomic techniques have found differential expression of different proteins [99, 100]. Numerous investigations have pointed out that a low number of PRPs is associated with an increased risk of dental caries [101, 102]. On the other hand, different studies have shown that the presence of mucins in patients with caries was significantly higher than in patients without this pathology [103]. Regarding other proteins (i.e., agglutinins, amylase, lactoferrin, and lysozyme), the results have been disparate and contradictory. Finally, in relation to salivary antibacterial peptides, there are contradictory results regarding their diagnostic value (i.e., alpha-defensins, cathelicidins, histatins, and staterins) [104, 105].

### 2.3. Diseases of the Oral Mucosa

Table 2 summarizes the use of protein-based techniques for salivary biomarker identification in oral mucosa diseases.

#### 2.3.1. Recurrent Aphthous Stomatitis

Recurrent aphthous stomatitis (RAS) is accompanied by recurrent oral ulcerations, commonly called aphthae [106]. Approximately 20% of the general population suffers from RAS [107]. Several reports have investigated the salivary proteome of patients suffering from this pathology. In particular, the most studied molecules have been cortisol, the OE-related peptides, Igs, and certain cytokines.

Different ELISA-based reports have found higher cortisol levels in patients with RAS than healthy controls [108, 109]. It has been hypothesized that these altered levels may be linked to the stress and anxiety present in these patients, establishing a neurobiological basis for this pathology. Total antioxidant capacity (TAC) is not related to the aetiology of this pathology; however, patients with RAS do tend to have altered levels of molecules related to OS [110–112]. Numerous studies have shown that levels of IgA and IgG increase considerably in RAS disease outbreaks [113]. Different inflammatory mediators, especially cytokines, can stimulate the production of MHC class I and II antigens in epithelial cells [106]. These cells trigger a cytotoxic response in T lymphocytes causing ulceration. In relation to this etiopathogenic model, numerous cytokines are found in greater amounts in patients with RAS (i.e., TNF-*α*, PGE2, VEGF, and IL-6) [114–116].

#### 2.3.2. Pemphigus and Pemphigoid

Vesiculobullous disorders are autoimmune-based pathologies characterized by the presence of antibodies against epithelial tissue-specific adhesion molecules. Its prevalence is 0.2 to 3 people out of every 100,000 [117].

Hallaji et al. demonstrated that by ELISA techniques, in the case of pemphigus, salivary desmoglein 1 and desmoglein 3 had sensitivities of 70% and 94%, respectively, in the diagnosis of this dermatological condition [118]. In the case of the pemphigoid, Esmaili et al. proved that the salivary concentration of BP180-NC16a is useful in the diagnosis of this disease [119]. It has also been shown that IgA and IgG salivary are markedly increased during pemphigoid and can be good alternatives in its diagnosis [120].

#### 2.3.3. Glossodynia or Burning Mouth Syndrome

The International Headache Society (IHS) defines burning mouth syndrome (BMS) as an intraoral burning or dysesthetic sensation, which is repeated daily for more than 2 hours/day for more than 3 months, without clinically evident causing lesions. BMS prevalence is barely 4% in the general population but reaches 18%–33% of postmenopausal women [121].

Due to the psychosomatic profile of this aetiology of this disease, stress-related proteins (such as cortisol and *α*-amylase) have been related to its presentation [122, 123]. There are few studies investigating the role of salivary Igs in this pathology, and the existing ones have contradictory results. Regarding cytokine-based investigations, the results are also contradictory for a large number of proteins (i.e., IL-1*β*, IL-6, IL-8, and TNF-*α*) [124–126].

Recently, nontargeted proteomic techniques have discovered other novel biomarkers for this pathology. A recent case-control study based on the LC-MS/MS and iTRAQ found 50 altered proteins (39 overexpressed and 11 subexpressed); three of them were validated through ELISA: alpha-enolase, IL-18, and KLK13 [127].

### 2.4. Oral Cancer and Potentially Malignant Oral Lesions

Table 3 summarizes the use of protein-based techniques for salivary biomarker identification in oral cancer and potentially malignant disorders.

#### 2.4.1. Oral Lichen Planus

Oral lichen planus (OLP) is a relatively common mucocutaneous disorder. OLP is originated through a chronic inflammation triggered by the epithelial cells apoptosis mediated by autocytotoxic T lymphocytes. According to the World Health Organization (WHO), OLP is considered an oral potentially malignant oral disorder (OPMD). There are several prospective long-term studies that show a malignant transformation rate of 1% over a 5-year average period [128]. Despite the progress of molecular biology in recent decades, there is no useful biomarker in assessing the risk of malignant transition of this entity; however, recent research based on salivary proteome analysis may be a step forward. The protein-based biomarkers most widely investigated in relation to the diagnosis of OLP have been cortisol, OS-related molecules, Igs, and cytokines.

In relation to cortisol, numerous investigations have investigated the relationship between psychological status and levels of this hormone in patients with OLP. Some case-control studies suggest that the elevated levels of this glucocorticoid are common among affected individuals [129, 130]. However, some reports do not find significant differences [131] or even find lower cortisol levels in OLP-affected patients [132]. Theoretically, cortisol generates a reduction in the number of lymphocytes and other immune cells and also dysfunctions in the hypothalamus-pituitary-adrenal (HPA) axis which trigger reduction in its production [133]. Lopez-Jornet et al. demonstrated that the levels of adiponectin were higher in OLP patients. In relation to Igs analysed via ELISA, IgA and IgG are considerably increased in patients with OLP compared to controls [129].

OLP aetiology is based on an imbalance between Th1/Th2 lymphocytes. The proinflammatory mediators that justify this imbalance are significantly increased in OLP-affected patients: IL-4, IL-10, IL-18, TNF-*α*, NF-*κ*B-related cytokines, CD44, and CD14 [113]. Interestingly, treatment with immunosuppressants such as corticosteroids or nonantibiotic macrolides and alternative therapies such as plant extracts and polyphenols have shown a relevant reduction in these inflammation-based biomarkers [133–135]. It should be noted that no research has yet provided a valid salivary biomarker to predict OLP malignant transformation.

Recently, nontargeted proteomic studies based on MS-based studies have provided new perspectives regarding the aetiology and diagnosis of the OLP [136, 137].

#### 2.4.2. Oral Leukoplakia

Oral leukoplakia (OL) is defined as “a white plaque of questionable risk having excluded (other) known diseases or disorders that carry no increased risk for cancer” [138]. The pooled estimated prevalence rate of OL varies between 1.7 and 2.7% in general population. OL is considered by the WHO as OPMD. Malignant transformation of oral leukoplakia in annual average is 1%. Despite the molecular biology progress to date, there is no certain marker to predict OL malignant transformation.

Proteomic studies focused on saliva to anticipate this malignancy are scarce, and the study of cytokines is based on ELISA techniques (i.e., IL-6, IL-8, and TNF-*α*) [139, 140]. Other reported proteins that were also useful to discern between OL and oral squamous cell carcinoma have been C4d, MDA, endothelin-1, and lactate dehydrogenase [141, 142]. Camisasca et al. recently reported that in a 2-DE gel-based proteomic study, 22 spots are much more abundant in patients with OL than in controls. One spot corresponded to CK10. Later, the authors validated this marker by means of immunohistochemistry [143].

#### 2.4.3. Oral Squamous Cell Carcinoma

Oral squamous cell carcinoma (OSCC) is the eighth most common cancer worldwide. Oral carcinogenesis is modulated by environmental and genetic factors [144]. The most extensively modifiable risk factors for this entity are tobacco and alcohol consumption [145, 146]. In the last 20 years, the study of HPV as a carcinogenic factor has also taken on strength [147]. The most extensively described OSCC-related modifiable risk factors are tobacco and alcohol consumption. In the last 20 years, the study of HPV as a carcinogenic factor has also raised. Despite all efforts on the side of public health and transnational research, a significant improvement in the 5-year survival rate of this neoplasm has not been achieved [144].

In relation to oral diseases, OSCC is by far the one in which proteomics research has employed its greatest efforts. A recent meta-analysis suggested that the use of simple or combined salivary biomarkers for the OSCC may be useful for diagnostic purposes [148]. One of the first family of proteins that aroused interest as OSCC biomarkers was interleukins family; in this sense, there are a large number of studies that ascertained their concentrations in saliva. Specifically, the most studied interleukins have been IL-6, IL-8, IL-1, and TNF-*α*. High levels of these proteins in saliva have been associated with OSCC. The biological plausibility of these high levels is found in the proangiogenic and proinflammatory functions of these analytes [149]. Elevated levels of IgG have also been detected in OSCC-affected patients versus controls, which ascertains the pivotal role of angiogenesis in oral carcinogenesis [150]. On the other hand, by means of ELISA techniques, Shpitzer et al. found that the salivary levels of Ki-67 and Cyclin D1 were also altered in these patients [151]. These findings are compatible with numerous immunohistochemistry reports in OSCC [152]. On the other hand, different investigations mainly based on Western blot, or MRS-based targeted proteomics techniques have found cell-surface glycoproteins overexpressed in patients with OSCC such as CD44, CD59, or CEA. Other biomarkers related to the zinc finger protein family (ZNF) such as ZNF510, Cyfra 21-1, and CK19 have also been reported [153–155]. In this sense, Jou et al. reported a sensitivity and specificity greater than 95% for salivary ZNF510 in the discrimination of tumors in early stages (T1 + T2) vs. advanced stages (T3 + T4) [156].

Nontargeted proteomic techniques have provided other unique proteins or panels useful as oncological markers. Hu et al. reported in a ROC curve analysis that a panel consisting of 5 proteins (M2BP, MRP14, CD59, catalase, and profilin) had a sensitivity of 90% and a specificity of 83% in the diagnosis of the OSCC via LC-MS/MS [155]. A Taiwanese group composed another panel with 4 proteins (MMP1, KNG1, ANXA2, and HSPA5) that able to diagnose OSCC and also to predict OPMDs malignant transformation [157]. Csosz et al. failed to validate some of the biomarkers described by other authors; this Hungarian group justified this fact by the ethnic and geographical variability of the target populations [158].

## 3. Conclusion and Future Perspectives

The advance in the field of salivanomics is a teragnostic revolution in oral pathology. The salivary proteome has a Janus role in oral pathology; oral proteins can provide cytoprotective functions in many of the oral diseases, and, at the same time, they can contribute to inflammation, infection, and even tumorigenesis in this cavity. In this sense, salivary proteome plays a pivotal role in oral homeostasis; imbalances at immunological and nonimmunological salivary defence systems can cause a myriad of possible mechanisms leading to oral pathologies.

Moreover, the salivary proteome is an immense source of useful biomarkers in the diagnosis and prognosis of this burden of diseases. However, the precise mechanisms underlying the role of oral proteins in the initiation and progression of these conditions are still largely unknown. Further research and a standardization of the analytical processes involved in its study are necessary to give a step forward. The study of the salivary proteome will mean an inexorable change in current dentistry.

## Figures and Tables

**Table 1:** Use of protein-based techniques for biomarkers identification in dental and periodontal diseases mucosa diseases.

Base disease	Number of patients	Age of patients	Matrix	Analytical technique	Determined parameter	Range of concentrations	Endpoints	Reference
Gingivitis	12	AR: 22–37	Unstimulated parotid saliva	ELISA	IgA	NR	↑IgA in patients as a response to experimental gingivitis	[56]
Gingivitis	20 (10 controls, 10 cases)	M: 35.6	USWS	2-DE and MALDI-TOF-MS	Total proteins	RQ	↑Blood-related proteins, immunoglobulin peptides, and keratins in gingivitis patients vs. controls	[57]
Pregnancy-associated gingivitis	54 (30 cases, 20 controls)	M: 29.3	SWS	ELISA	hBD-1, hBD-2, and HNP-1	^∗^	↑hBD-1, ↑hBD-2, and ↑HNP-1 in pregnancy-related gingivitis patients vs. controls	[62]
Pregnancy-associated gingivitis	178 (78 cases, 69 controls)	NR	USWS	ELISA	IL-1*β*, and ANXA1	Pregnant: IL-1*β* (566.0 ± 445.1 pg/ml); ANXA1 (1184 ± 1495 pg/ml). Control: IL-1*β* (258.3 ± 225.0 pg/ml); ANXA1 (495.2 ± 990.9 pg/ml)	↑IL-1*β* and ANXA1 in pregnancy-related gingivitis patients vs. controls	[64]
Pregnancy-associated gingivitis	176 (59 pregnant, 47 postpartum, and 70 controls)	AR: 18–48	USWS	ELISA	PGE2 and TNF-*α*	Pregnant: PGE2 (505.6 ± 198.5 pg/ml); TNF-*α* (61.21 ± 11.75 pg/ml). Postpartum: PGE2 (621.7 ± 191 pg/ml); TNF-*α* (72.96 ± 58.77 pg/ml). Control group: PGE2 (569.2 ± 297.7 pg/ml); TNF-*α* (76.67 ± 33.54 pg/ml)	↑TNF-*α* in patients with pregnancy-induced gingivitis vs. postpartum and controls	[65]
Gingivitis	35 (10 controls, 25 cases)	M: 13.6	USWS	ELISA	Cystatin C, IL-1*β*, and TNF-*α*	Control: cystatin C (3.721 ± 2.1740 mg/ml); IL-1*β* (2.270 ± 1.595 pg/ml); TNF-*α* (623.386 ± 395.101 pg/ml). Case: cystatin C (2.987 ± 1.139 mg/ml); IL-1*β* (5.587 ± 5.441 pg/ml); TNF-*α* (321.163 ± 335.043 pg/ml)	No significant alterations	[66]
ChP	46 (21 controls, 25 cases)	M: 53	USWS	ELISA	IgA, IgG, IgE, and IgM	Controls: IgA (596.9 ± 103.6 ng/l), IgG (369.6 ± 44.6 ng/l), IgE (0.24 ± 0.89 IU/ml), IgM (779 ± 49.5 ng/ml). Case: IgA (670 ± 110.0 ng/ml), IgG (370 ± 60.9 ng/ml), IgE (0.20 ± 1.0 IU/ml), IgM (791.4 ± 43.7 ng/ml)	↑IgA in chronic periodontitis patients vs. controls	[70]
ChP	70 (20 controls, 50 cases)	AR: 18–45	USWS	ELISA	IgA, IL-1*β*, and MMP-8	Control: IgA (81.23 ± 24.61 *μ*g/ml); IL-1*β* (89.93 ± 25.48 pg/ml); MMP-8 (57.95 ± 31.64 ng/ml). Case: IgA (196.48 ± 54.61 *μ*g/ml); IL-1*β* (530.76 ± 343.85 pg/ml); MMP-8 (672.18 ± 411.0 ng/ml)	↑IgA, ↑IL-1*β*, and ↑MMP-8 in chronic periodontitis patients vs. controls	[71]
Aggressive periodontitis	10 (5 controls and 5 cases)	M: 24	USWS	2-DE and LC-MS/MS validation via ELISA	Total proteins	RQ	↓Lactoferrin, ↑IgA2, and ↑albumin in aggressive periodontitis patients vs. controls	[74]
ChP	20 (10 controls and 10 cases)	AR: 26–50	USWS	2-DE and MALDI-TOF-MS	Total proteins	RQ	↑*α*-Amylase variants in chronic periodontitis patients vs. controls	[75]
ChP	57 (38 obese patients (13 periodontitis and 33 nonperiodontitis) and 19 healthy patients)	AR: 35–65	SWS	SELDI-TOF-MS	Total proteins	RQ	↑Albumin, ↑a and b hemoglobin, ↑ *α*-defensins 1, 2, and 3 in periodontitis obese patients vs. controls	[76]
ChP	25 (15 periodontitis patients with type two diabetes and 10 controls)	AR: 40–60	USWS	2-DE and LC-MS/MS	Total proteins	RQ	↑Immunoglobulin J chain, ↓polymeric immunoglobulin receptor, ↑plastin-2, ↓actin-related protein, ↓interleukin-1 receptor antagonist, and ↑leukocyte elastase chronic periodontitis patients vs. controls	[77]
ChP and gingivitis	67 (17 chronic periodontitis, 17 gingivitis, and 33 healthy)	AR: 20–64	USWS	LC-MS/MS validation via MRM	Total proteins	RQ	↑Matrix metalloproteinase-9, ↑Ras-related protein-1, ↑actin-related protein 2/3 complex subunit 5, ↓clusterin, and ↓deleted in malignant brain tumors 1 in chronic periodontitis and gingivitis patients vs. controls	[78]
ChP	54 (20 controls, 43 cases)	AR: 20–73	USWS	ELISA	IL-1*β*, IL-1ra, platelet-derived growth factor-BB, VEGF, MMP-8, MMP-9, CRP, and lactoferrin	Control: MMP-8 (36.8 (16.9–295.5 ng/ml)); lactoferrin (10,877 (5808–20,937 ng/ml)). Case: MMP-8 (203.7 (86.8–609.2 ng/ml)); lactoferrin (10,877 15,801 (12707–18,687 ng/ml))	↑IL-1*β* and ↑MMP-8 in chronic periodontitis and gingivitis patients vs. controls	[80]
ChP	46 (20 controls, 26 cases)	M: 49	USWS	ELISA	HGF	Control: HGF (0.06 to 5.38 ng/ml). Case: HGF (0.68 ng/ml (range: 0–7.33))	↑HGF in chronic periodontitis patients vs. controls	[83]
ChP	52 (24 controls, 28 cases)	AR: 20–50	USWS	ELISA	IL-1*β*	Control: IL-1*β* (161.51 pg/ml). Case: IL-1*β* (1312.75 pg/ml)	↑IL-1*β* in chronic periodontitis patients vs. controls	[84]
ChP	42 (15 controls, 27 cases)	AR: 35–55	SWS	ELISA	MMP-8, MMP-9, TIMP-1, TIMP-2, and MPO	NR	↑MMP-8, ↑TIMP-1, and ↑MPO in chronic periodontitis patients vs. controls	[88]
Orthodontically induced inflammatory root resorption	72 (24 controls, 48 cases)	AR: 10–30	USWS	2-DE and LC-MS/MS validation via Western blot	Total proteins	RQ	↑P21-ARC and ↑CDC42 in orthodontically induced inflammatory root resorption vs. controls	[91]
Caries	32 (16 controls, 16 cases)	AR:18–29	USWS	2-DE and LC-MS/MS	Total proteins	RQ	↑Amylase, ↑IgA, and ↑lactoferrin, ↓cystatins, ↓acidic PRPs, and ↓lipocalin-1 in dental caries patients vs. controls	[99]
Caries	30 (10 controls, 20 cases)	AR: 10–12	USWS	iTRAQ-LC MS/MS validation via MRM	Total proteins	RQ	↑Mucin 7, ↑mucin 5B, ↑histatin 1, ↑cystatin S, ↑cystatin SN, and ↑basic salivary ↑proline-rich protein 2 in dental caries patients vs. controls	[100]
Caries	100 (50 controls, 50 cases)	AR: 4–6	USWS	2-DE	Total proteins	RQ	↑Proline-rich protein bands in dental caries patients vs. controls	[101]
Caries	26 (13 controls, 13 cases)	AR: 3–4	SWS	MALDI-TOF MS combined with magnetic beads	Total proteins	RQ	↑2 specific peptides with m/z values 3162.0 Da and 3290.4 Da in caries patients vs. controls	[104]
Caries	30 (10 controls, 20 cases)	AR: 4.7	SWS	MALDI-TOF MS combined with magnetic beads	Total proteins	RQ	↑Histatin-1 in caries patients vs. controls	[105]

Abbreviations: 2-DE: two-dimensional gel electrophoresis; ANXA: annexin; AR: age range; ARC: activity-regulated cytoskeleton-associated protein; CDC: cell-division cycle protein; ChP: chronic periodontitis; CRP: c-reactive protein; ELISA: enzyme-linked immunosorbent assay; HDB: hemoglobin subunit delta; HGF: hepatocyte growth factor; HNP: human neutrophil peptide; Ig: immunoglobulin; IL: interleukin; ITRAQ: isobaric tags for relative and absolute quantitation; LC-MS/MS: liquid chromatography tandem-mass spectrometry; M: mean; NR: not reported; MALDI-TOF: matrix-assisted laser desorption/ionization; MMP: matrix metalloproteinase; MPO: myeloperoxidase; MRM: multiple reaction monitoring; PG: prostaglandin; PRP: proline-rich protein; RQ: relative quantification; SWS: stimulated whole saliva; TIMP: tissue inhibitor of metalloproteinases; TNF: tumor necrosis factor; UWSW: unstimulated whole saliva; VEGF: vascular endothelial growth factor. ^∗^for additional data see original source.

**Table 2:** Use of protein-based techniques for biomarkers identification in oral mucosa diseases.

Base disease	Number of patients	Age of patients	Matrix	Analytical technique	Determined parameter	Range of concentrations	Endpoints	Reference
RAS	30 (10 controls, 20 cases)	M: 35.9	USWS	ELISA	Cortisol	Control: cortisol (0.64 ± 0.36 mg/dl); case: cortisol (0.57 ± 0.25 mg/dl)	↑Cortisol in recurrent aphthous stomatitis patients vs. controls	[108]
RAS	68 (34 controls, 34 cases)	M: 23.29	USWS	ELISA	Cortisol and amylase	Control: cortisol (3.65 ± 2.5 ng/dl); amylase (128.74 ± 86.3 U/ml); case: cortisol (3.35 ± 1.8 ng/dl); amylase (155.09 ± 116.1 U/ml)	No significant differences	[109]
RAS	75 (25 controls, 50 cases)	M: 27.5	USWS	ELISA	MPO	Control: MPO (21.36 ± 14.73 U g^−1^); case: MPO (19.22 ± 18.97 U g^−1^).	No significant differences	[111]
RAS	62 (30 controls, 32 cases)	AR: 14–46	Unstimulated parotid saliva	ELISA	Superoxide dismutase, glutathione peroxidase, and catalase	Control: superoxide dismutase (0.56 ± 0.11 U/mg protein); catalase (0.78 ± 0.03 U/mg); glutathione peroxidase (2.88 ± 0.18 U/mg). Case: superoxide dismutase 0.90 ± 0.04 (U/mg); catalase (0.90 ± 0.04 U/mg); glutathione peroxidase (1.70 ± 0.1 U/mg).	↑Superoxide dismutase, ↑glutathione peroxidase, and ↓catalase in recurrent aphthous stomatitis patients vs. controls	[112]
RAS	52 (26 controls, 26 cases)	AR: 22–64	USWS	ELISA	IL-6 and TNF-*α*	Control: IL-6 (9.38 ± 9.23 pg/ml); TNF-*α* (7.88 ± 8.45 pg/ml); case: IL-6 (12.5 ± 17.51 pg/ml); TNF-*α* (28 ± 26.19 pg/ml).	↑IL-6 in recurrent aphthous stomatitis vs. controls	[114]
Behçet's disease and RAS	119 (60 controls and 59 cases (33 Behçet's disease and 16 recurrent aphthous stomatitis))	AR: 16–45	USWS	ELISA	Salivary epidermal growth factor	Control: salivary epidermal growth factor (2758.7 ± 81,657.9 pg/ml). Behçet's disease: salivary epidermal growth factor (1939.7 ± 81,561.5 pg/ml). Recurrent aphthous stomatitis: salivary epidermal growth factor (1650.5 ± 8704.7 pg/ml)	↓Salivary epidermal growth factor in recurrent aphthous stomatitis, and Behçet's disease vs. controls	[115]
Pemphigus vulgaris	127 (77 controls, 50 cases)	M: 46.84	USWS	ELISA	Desmoglein 1 and 3	Control: all controls were below cut-off value; case: desmoglein 1 (58.25 ± 47.52 index value); desmoglein 3 (144.47 ± 53.42 index value)	↑Desmoglein 1 and ↑3 in pemphigus vulgaris patients vs. controls	[118]
BP	100 (50 controls, 50 cases)	AR: 38–91	USWS	ELISA	BP180 NC16a and BP230-C3	NR	BP180 NC16a useful as diagnostic marker for pemphigoid	[119]
Mucous membrane pemphigoid	114 (50 controls, 50 cases)	AR: 26–87	USWS and stimulated parotid saliva	ELISA	IgG and IgA	NR	IgA useful as diagnostic marker for pemphigoid	[120]
BMS	60 (30 controls, 30 cases)	M: 63.8	USWS	ELISA	Cortisol and *α*-amylase	Control: cortisol (3.69 ± 3.07 ng/ml); amylase (146.22 ± 130.4 IU/l); case: cortisol (4.50 ± 3.68 ng/ml); amylase (351.68 ± 142.5 IU/l)	↑Cortisol and ↑*α*-amylase in BMS patients vs controls	[122]
BMS	29 (14 controls, 15 cases)	M: 65.7	USWS and SWS	ELISA	Cortisol, 17b-estradiol, progesterone, dehydroepiandrosterone, and *α*-amylase	^∗^	↑Cortisol in USWS, and of ↑17b-estradiol in SWS in BMS vs. controls	[123]
BMS	270 (90 controls, 180 cases)	AR: 15–88	USWS	ELISA	Albumin, lysozyme, amylase, IgM, IgG, and IgA	Control: albumin (9.36 ± 3.44 mg/dl); lysozyme (24.03 ± 3.38 mg/ml); amylase (1638.0 ± 372.0 IU/l); IgM (1.02 ± 0.06 mg/dl); IgG (0.79 ± 0.15 mg/dl); IgA (24.34 ± 1.26 mg/dl). Case: albumin (18.04 ± 2.56 mg/dl); lysozyme (28.10 ± 3.48 mg/dl); amylase (3030.0 ± 470.0 IU/l); IgM (2.19 ± 0.68 mg/dl); IgG (4.47 ± 0.76 mg/dl); IgA (33.15 ± 3.53 mg/dl)	↑Albumin, ↑IgA, ↑IgG, ↑IgM, and ↑lysozyme in BMS patients vs. controls	[124]
BMS	45 (30 controls, 15 cases)	M: 55.2	USWS	ELISA	IgA	Control: IgA (164.71 ± 158.80 *μ*g/ml). Case: IgA (176.14 ± 97.23 *μ*g/ml).	No significant differences	[125]
BMS	97 (50 controls, 47 cases (BMS, oral lichen planus, and RAS))	NR	USWS	ELISA	IgE	Control: IgE (20.6 ± 66.6 mg/dl). Case: IgE (8.07 ± 30.4 mg/dl)	No significant differences	[126]
BMS	38 (19 controls, 19 cases)	NR	USWS	LC-MS/MS validation via ELISA	Total proteins	RQ	↑Alpha-enolase, ↑IL-18, and ↑KLK13 in BMS vs. controls	[127]

Abbreviations: AR: age range; BMS: burning mouth syndrome; BP: bullous pemphigoid; ELISA: enzyme-linked immunosorbent assay; Ig: immunoglobulin; IL: interleukin; KLK: kallikrein-related peptidase; LC-MS/MS: liquid chromatography tandem-mass spectrometry; M: mean NR: not reported; MPO: myeloperoxidase; NC: noncollagenous; RAS: recurrent aphthous stomatitis; RQ: relative quantification; SWS: stimulated whole saliva; TNF: tumor necrosis factor; UWSW: unstimulated whole saliva. ^∗^for additional data see original source.

**Table 3:** Use of protein-based techniques for biomarkers identification in oral cancer and potentially malignant disorders.

Base disease	Number of patients	Age of patients	Matrix	Analytical technique	Determined parameter	Range of concentrations	Endpoints	Reference
OLP	65 (32 controls, 33 cases)	M: 57	USWS	ELISA	Cortisol, IgA, and adiponectin	Control: IgA (48.9 ± 32.8 mg/l); cortisol (0.4 ± 0.2 *μ*g/dl); adiponectin (20.1 ± 24.9 mg/ml). Case: IgA (80.3 ± 51.3 mg/l); cortisol (0.5 ± 0.3 *μ*g/dl); adiponectin (38.2 ± 63.5 mg/ml)	↑Cortisol and ↑IgA in OLP patients vs. controls	[129]
OLP	61 (31 controls, 30 cases)	M: 54	USWS	ELISA	Cortisol	Control: cortisol (5.21 ± 2.54 mg/ml). Case: cortisol (4.67 ± 0.33 mg/ml)	No significant differences	[130]
OLP	62 (31 controls, 31 cases)	M: 30	USWS	ELISA	Cortisol and dehydroepiandrosterone	Control: cortisol (14.10 [8.60–18–30] nmol/l); dehydroepiandrosterone (0.66 (0.51–1.22) nmol/l); case: cortisol (13.50 (10.50–21–30), nmol/l); dehydroepiandrosterone (0.75 (0.46–0.99) nmol/l)	No significant differences	[131]
OLP	20 (10 controls, 10 cases)	M: 58.1	Unstimulated parotid saliva	ELISA	Cortisol	^∗^	No significant differences	[132]
OLP	20 (10 controls, 10 cases)	M: 57	USWS	LC-MS/MS validation via MRM	Total proteins	RQ	↑S100A8, ↑S100A9, and ↑haptoglobin in OLP vs. controls	[136]
OLP	12 (6 controls, 6 cases)	AR: 23–60	USWS	2-DE and MALDI-TOF-MS	Total proteins	RQ	↑Urinary prokallikrein and ↓PLUNC in OLP vs. controls	[137]
OL and OSCC	88 (31 controls, 29 OL, 28 OSCC)	M: 60.89	USWS	ELISA	IL-1*β*, IL-6, and TNF-*α*	Control: IL-1*β* (354 ± 61.51 pg/ml); IL-6 (16 ± 3.91 pg/ml); TNF-*α* (38 ± 3.23 pg/ml). OL: IL-1*β* (143 ± 54.74 pg/ml); IL-6 (18 ± 5.19 pg/ml); TNF-*α* (30 ± 3.01 pg/ml). OSCC: IL-1*β* (906 ± 62.21 pg/ml); IL-6 (129 ± 66.59 pg/ml); TNF-*α* (34 ± 21.58 pg/ml)	↑IL-1*β* and ↑IL-6 in OL and OSCC patients vs. controls	[139]
OL and OSCC	75 (25 controls, 25 OL/oral submucosa fibrosis (OPMDs), 25 oral cancer)	M: 53.2	USWS	ELISA	IL-8	Control: IL-8 (210.096 ± 142.302 pg/ml). OL/oral submucosa fibrosis: IL-8 (299.513 ± 158.165 pg/ml). OSCC: IL-8 (1718.610 ± 668.294 pg/ml)	↑IL-8 in OSCC patients vs. controls and OL	[140]
OL and OSCC	110 (48 OL, 62 OSCC)	NR	USWS	ELISA	C4d	OL: Cd4 IL-8 (0.04 ± 0.03 *μ*g/ml^−1^). OSCC: Cd4 (0.07 ± 0.07 *μ*g/ml^−1^)	↑C4d in OSCC patients vs. OL patients	[141]
OL and OSCC	69 (20 OSCC, 15 OSCC cured, 15 OL, and 20 controls)	AR: 32–89	USWS	ELISA	Endothelin-1	NR	No significant differences	[142]
OL	25 (10 controls, 15 cases)	M: 73.8	USWS	2-DE and MALDI-TOF-MS	Total proteins	RQ	↓Apolipoprotein A-1 and ↑cystatin SN precursor in OL patients vs. controls	[143]
Head and neck cancer	24 (8 controls, 16 cases)	M: 51.88	USWS	2-DE and LC-MS/MS	Total proteins	RQ	↑Beta fibrin, ↑S100 calcium-binding protein, ↑transferrin, ↑ immunoglobulin heavy chain constant region gamma, ↑cofilin-1 and, ↓transthyretin in Head and neck cancer patients vs. controls	[150]
OSCC	38 (19 controls, 19 cases)	M: 66	USWS	ELISA	Maspin, phosphor Src, CycD1, Ki67, MMP-9, and LDH	^∗^	↓Maspin, ↓phospho Src, ↑CycD1, ↑Ki67, ↑MMP-9, and ↑LDH in OSCC patients vs. controls	[151]
OSCC	53 (26 controls, 27 cases)	M: 53	USWS	ELISA	ErbB2 and CEA	Control: ErbB2 (4.9 ± 2.1 ng/ml); CEA (22.6 ± 22.1 ng/ml); Case: ErbB2 (5.2 ± 1.8 ng/ml); CEA (42.6 ± 21.1 ng/ml)	↑CEA in OSCC patients vs. controls	[153]
OSCC	54 (25 controls, 29 cases)	M: 61.9	USWS	LC-MS/MS validation via ELISA	IL-1*α*, IL-1*β*, IL-6, IL-8, TNF-*α*, VEGF, catalase, profilin-1, S100A9, CD59, galectin-3-bindig protein, CD44, thioredoxin, and keratin-19	^∗^	↑S100A9 and ↑IL-6 in OSCC patients vs. controls	[154]
OSCC	128 (64 controls, 64 cases)	M: 54	USWS	2-DE and LC-MS/MS validation via ELISA	Total proteins	RQ	↑M2BP, ↑MRP14, ↑CD59, ↑catalase, and ↑profilin in OSCC patients vs. controls	[156]
OSCC	77 (30 controls, 47 cases)	M: 53.3	USWS	MALDI-TOF MS combined with magnetic beads validation via ELISA	Total proteins	RQ	↑24-mer ZNF510 peptide in OSCC vs. controls	[156]
OSCC	460 (96 controls, 103 low-risk OPMDs, 130 high-risk OPMDs, and 131 OSCC)	M: 50.7	USWS	LC–MS/MS validation via MRM	Total proteins	RQ	↑MMP1, ↑KNG1, ↑ANXA2, and ↑HSPA5 in OSCC patients vs. controls	[157]
OSCC	37 (17 controls, 20 cases)	M: 57	USWS	LC–MS/MS validation via ELISA	Total proteins	RQ	No significant differences	[158]

Abbreviations: 2-DE: two-dimensional gel electrophoresis; ANXA: annexin; AR: age range; C4d: complement component 4; CD: cell-surface protein; CEA: carcinoembryonic antigen; CycD1: cyclin D1; ELISA: enzyme-linked immunosorbent assay; erbB-2: human epidermal growth factor receptor 2; HSP: heat shock protein; Ig: immunoglobulin; IL: interleukin; KNG: kininogen; LC-MS/MS: liquid chromatography tandem-mass spectrometry; LDH: lactate dehydrogenase; M2BP: human Mac-2-binding protein; M: mean; MALDI-TOF: matrix-assisted laser desorption/ionization; Maspin: mammary serine protease inhibitor; MMP: matrix metalloproteinases; MRP: migration inhibitory factor-related protein; NR: not reported; OL: oral leucoplakia; OLP: oral lichen planus; OPMD: oral potentially malignant disorder; OSCC: oral squamous cell carcinoma; phosphor-SRC: phosphorylated SRC; PLUNC: palate, lung, and nasal epithelium clone protein; RQ: relative quantification; SWS: stimulated whole saliva; TNF: tumor necrosis alpha; UWSW: unstimulated whole saliva; VEGF: vascular endothelial growth factor; ZNF: zinc finger protein. ^∗^for additional data see original source.
